# Pneumocystis Pneumonia Mimicking Atypical Pneumonia in a Patient With Human Immunodeficiency Virus Infection

**DOI:** 10.7759/cureus.28388

**Published:** 2022-08-25

**Authors:** Takehiro Hashimoto, Masaru Ando, Shinichi Nureki, Kosaku Komiya, Kazufumi Hiramatsu

**Affiliations:** 1 Department of Respiratory Medicine and Infectious Diseases, Oita University Faculty of Medicine, Yufu, JPN

**Keywords:** ground-glass opacities, atypical pneumonia, azithromycin, hiv, pneumocystis pneumonia

## Abstract

We report a case of pneumocystis pneumonia (PCP) that mimicked atypical pneumonia in a patient with human immunodeficiency virus (HIV) infection. A 44-year-old Japanese man with persistent fever and dyspnea for a month was diagnosed with atypical pneumonia because of bilateral ground-glass opacities on chest computed tomography (CT). Ground-glass opacities on chest CT diminished with three days treatment of azithromycin; however, his symptoms were persistent. Final diagnosis of HIV and PCP infection was eventually confirmed. Physicians should consider the possibility of PCP even when pulmonary manifestations resolve with azithromycin in patients with HIV infection.

## Introduction

The most common manifestations of pneumocystis pneumonia (PCP) are subacute onset of progressive dyspnea, fever, non-productive cough, and chest discomfort that worsens within days to weeks. The most common finding of chest computed tomography (CT) of PCP is extensive ground-glass attenuation that may be patchy or diffuse and with a central, perihilar, and upper lobe predominance [1]. PCP is usually seen in immunocompromised hosts with human immunodeficiency virus (HIV) infection, malignancies, and on drug therapies such as chemotherapy and steroids [2]. On the other hand, clinical symptoms of atypical pneumonia are low-grade fever, dry cough, and malaise, and chest CT typically demonstrates focal ground-glass opacity in a lobular distribution, which is often diffuse and bilateral [3-4]. Therefore, clinical diagnosis of PCP associated with HIV infection is difficult because its manifestations are very non-specific and similar to those of atypical pneumonia unless the clinicians suspect the patient as having HIV infection.

Trimethoprim-sulfamethoxazole (TMP-SMX) (TMP 15mg/kg/day) is the first-line regimen for the treatment of mild-to-severe PCP. Intravenous pentamidine, which is as effective as TMP-SMX, is an alternative to TMP-SMX; however, the incidence of adverse events is higher compared to TMP-SMX. Atovaquone, which is less effective but better tolerated than TMP-SMX, is the oral alternative regimen for mild and moderate PCP. Adjunctive corticosteroid therapy is recommended for moderate to severe PCP (arterial oxygen pressure less than 70 torr on room air and an alveolar-arterial oxygen gradient greater than 35 torr) [5]. Although macrolides are not used for the treatment of PCP, in animal models, macrolides have activity against PCP in vivo [6]. Dunne et al. suggested that azithromycin is beneficial only as primary prophylaxis and not secondary prophylaxis against PCP [7].

We herein report a case of PCP in an HIV-infected patient that mimicked the chest CT findings of atypical pneumonia. The diffuse ground-glass opacity disappeared after the administration of azithromycin. Clinicians should consider the possibility of PCP even when pulmonary manifestations resolve with azithromycin in patients with HIV infection.

## Case presentation

**Figure 1:**
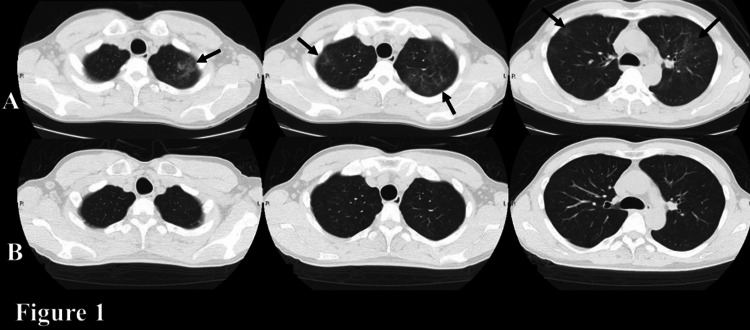
Section of axial chest CT. (A) Ten days prior to admission (in the figure above). (B) On admission (in the figure below). Chest CT revealed diffuse ground-glass opacity (GGO) (arrows) in (A) and improvement of GGO in (B).

A 44-year-old Japanese man with persistent fever and dyspnea was admitted to our hospital. He complained of fever and dry cough for a month. Ten days prior to admission, a chest X-ray showed normal findings, while chest CT revealed diffuse ground-glass opacity in the outpatient setting (Figure 1A). The patient was diagnosed with atypical pneumonia, and azithromycin (500mg, daily) was prescribed for three days. Two days prior to admission, his dyspnea and fever had not improved. He was a care worker. He had a history of homosexual activity. He was not on any medication and had no history of smoking and injecting drugs. A physical examination revealed a body temperature of 37.7°C, blood pressure of 116/72 mmHg, pulse of 93 beats/minutes, and SpO_2_ of 98% on room air. He had no cardiac murmurs, and his lungs were clear on auscultation. Other physical examinations revealed no remarkable findings. An arterial blood gas analysis revealed the following: pH of 7.45, PaO_2_ of 95 torr, and PaCO_2_ of 39 torr. His A-aDO2 (6.3 torr) was within normal range on room air. Laboratory findings revealed normal white blood cell count, mild anemia, elevated (1-3)-β-D-glucan (BDG), and elevated KL-6, and the following: white blood cell count of 3,060 cells/μL, hemoglobin of 11.6 g/dL, platelets of 1.6 x 105 cells/μL, albumin of 4.1 g/dL, C-reactive protein of 0.01 mg/dL, BDG of 96 pg/mL (normal: <11.0 pg/mL), and KL-6 of 576 U/mL (normal: ≤500 U/mL). An HIV screening test was positive, his HIV-RNA titer was 9.6 x 105 copies/mL, and his CD4+ cell count was 9 cells/μL. Ground-glass opacity on chest CT disappeared on admission (Figure 1B).

**Figure 2:**
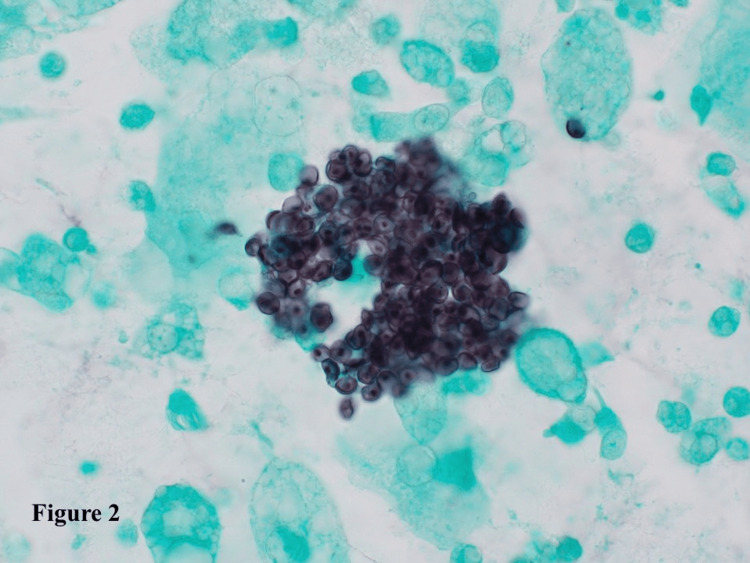
Grocott staining of BAL fluid. Grocott staining-positive cysts were observed in the BAL fluid. BAL, bronchoalveolar lavage

Despite the normal chest CT findings, bronchoalveolar lavage (BAL) was performed due to his persistent symptoms. Cytological finding of BAL cytology (Grocott staining) revealed clusters of *Pneumocystis jirovecii* (Figure 2).

*Pneumocystis jirovecii*-PCR test in BAL fluid was positive, and cytomegalovirus-PCR was negative. The patient was diagnosed with HIV/AIDS and PCP. TMP-SMX was initiated (TMP 15mg/kg/day divided Q8h) without adjunctive corticosteroid therapy. Low-grade fever and dry cough gradually improved on day 5. TMP-SMX was switched to atovaquone because of hyponatremia and thrombocytopenia on day 8. On day 15, serum BDG and KL-6 levels decreased to 54 pg/mL and 363 U/mL, respectively. Atovaquone was discontinued on day 21, and inhalation of pentamidine (300mg per month) was started as secondary prophylaxis against PCP. We started ART (emtricitabine [200mg]/tenofovir alafenamide [25mg], and dolutegravir [50mg]) on day 31. He was discharged on day 57. He is stable without recurrence of PCP for five years after discharge.

## Discussion

We described the case of a patient with PCP and HIV infection, with radiographic findings of diffuse ground-glass opacity, which mimicked atypical pneumonia and disappeared after the administration of azithromycin.

In our case, at first, the patient was misdiagnosed with atypical pneumonia. Clinical manifestations of atypical pneumonia are non-specific [3], and chest CT findings of atypical pneumonia include a differential diagnosis such as PCP [8]. In an outpatient setting, rapid antigen detection assays are used for diagnosis of atypical pneumonia; however, these tests have a lower diagnostic sensitivity than genetic diagnostic methods such as PCR [9]. Moreover, atypical pathogens are difficult to culture. For these reasons, empirical treatment is often performed with macrolide, fluoroquinolone, and tetracyclines for atypical pneumonia [10]. There is no serological diagnostic marker to confirm the diagnosis of PCP. BDG and KL-6 are used as serologic biomarkers in the adjunctive diagnosis of PCP, and this combination test has 94.3% sensitivity and 89.6% specificity [11], and the sensitivity of Grocott staining of sputum is low [12]. Furthermore, it may be difficult to perform BAL in an outpatient setting immediately. Thus, clinical diagnosis of both atypical pneumonia and PCP is often difficult.

We hypothesize that chest CT findings could improve in our case for the following reasons: (1) pneumonitis due to acute HIV infection, (2) spontaneous remission, and (3) efficacy of azithromycin. Four previous cases of pneumonitis due to acute HIV infection have been reported [13-16]. All improved spontaneously; however, the time required to improve ranged from two weeks to three months. In our case, envelope bands (gp160, gp120, and gp41) and gag gene p24 were detected by western blotting. Thus, we thought that our case was not pneumonitis due to acute HIV infection. Tanaka et al. reviewed 10 cases of PCP with spontaneous resolution (HIV-negative, n=9; HIV-positive, n=1) and suggested that the spontaneous resolution of PCP was associated with changes in the immune status due to underlying disease or the administration of immunosuppressive drugs [17]. Although the chest X-ray findings improved spontaneously three weeks after the diagnosis in an HIV-infected patient with spontaneous resolution of PCP, a confounding factor may have been involved because two doses of TMP-SMX were administered after the diagnosis [17-18].

To our knowledge, there are no reports on the improvement of chest CT findings of PCP from HIV patients with azithromycin. Macrolides might be useful for not only antimicrobial effects but also immunomodulatory effects [19]. Moreover, animal studies have revealed that the combination of sulfonamides and azithromycin prevents PCP; however, these drugs alone do not prevent PCP [6]. In patients with HIV infection receiving PCP prophylaxis, such as co-trimoxazole, dapsone, and pentamidine, weekly azithromycin-based regimen for MAC (*Mycobacterium avium* complex) prophylaxis provided an additional decrease in the risk of developing PCP and had no effect of secondary prevention of PCP. Thus, Dunne et al. suggested that azithromycin is beneficial only as primary prophylaxis and not secondary prophylaxis against PCP [7]. Moreover, 10-39% of patients with documented PCP may present with normal, near normal, or equivocal chest radiography [20]. Thus, the chest CT findings of PCP in HIV-infected patient with persistent symptoms might be the spontaneous resolution of ground-glass opacity rather than associated directly with azithromycin because basically the symptoms resolve with the treatment. If HIV infection is suspected, repeated sputum examinations or BAL should be considered for patients with persistent fever, even when the chest radiography findings improve with azithromycin, because the prevalence of PCP in the HIV-infected patients was reported around 40% in Japan.

## Conclusions

PCP may be initially misdiagnosed as atypical pneumonia if chest X-ray or chest CT demonstrates diffuse ground-glass opacity, especially in cases with no information about HIV infection. Even when the radiographic findings improve with conventional azithromycin therapy, PCP infection should be considered.

## References

[1] Oikonomou A, Prassopoulos P (2013). Mimics in chest disease: interstitial opacities. Insights Imaging.

[2] Arshad V, Iqbal N, Saleem HA, Irfan M (2017). Case of undiagnosed pneumocystis pneumonia (PCP). BMJ Case Rep.

[3] Johnson DH, Cunha BA (1993). Atypical pneumonias. Clinical and extrapulmonary features of Chlamydia, Mycoplasma, and Legionella infections. Postgrad Med.

[4] Miyashita N, Sugiu T, Kawai Y (2009). Radiographic features of Mycoplasma pneumoniae pneumonia: differential diagnosis and performance timing. BMC Med Imaging.

[5] Tasaka S (2015). Pneumocystis pneumonia in human immunodeficiency virus-infected adults and adolescents: current concepts and future directions. Clin Med Insights Circ Respir Pulm Med.

[6] Hughes WT (1991). Macrolide-antifol synergism in anti-Pneumocystis carinii therapeutics. J Protozool.

[7] Dunne MW, Bozzette S, McCutchan JA (1999). Efficacy of azithromycin in prevention of Pneumocystis carinii pneumonia: a randomised trial. California Collaborative Treatment Group. Lancet.

[8] Kishaba T (2016). Community-acquired pneumonia caused by mycoplasma pneumoniae: how physical and radiological examination contribute to successful diagnosis. Front Med (Lausanne).

[9] Miyashita N (2022). Atypical pneumonia: pathophysiology, diagnosis, and treatment. Respir Investig.

[10] Cunha BA (2006). The atypical pneumonias: clinical diagnosis and importance. Clin Microbiol Infect.

[11] Esteves F, Calé SS, Badura R (2015). Diagnosis of Pneumocystis pneumonia: evaluation of four serologic biomarkers. Clin Microbiol Infect.

[12] Turner D, Schwarz Y, Yust I (2003). Induced sputum for diagnosing Pneumocystis carinii pneumonia in HIV patients: new data, new issues. Eur Respir J.

[13] Jouveshomme S, Couderc LJ, Ferchal F, Vignon D, Autran B, Balloul E, Caubarrere I (1997). Lymphocytic alveolitis after primary HIV infection with CMV coinfection. Chest.

[14] Ong EL, Mandal BK (1991). Primary HIV-I infection associated with pneumonitis. Postgrad Med J.

[15] Longworth DL, Spech TJ, Ahmad M, Sharp DE, Fishleder AJ, Yen-Lieberman B, Proffitt MR (1990). Lymphocytic alveolitis in primary HIV infection. Cleve Clin J Med.

[16] Case Records of the Massachusetts General Hospital (1989). Weekly clinicopathological exercises. Case 33-1989. A 26-year-old woman with fever, diarrhea, leukopenia, thrombocytopenia, and hypoxemia. N Engl J Med.

[17] Tanaka Y, Saraya T, Kurai D, Ishii H, Takizawa H, Goto H (2014). Spontaneous resolution of Pneumocystis jirovecii pneumonia on high-resolution computed tomography in a patient with renal cell carcinoma. Am J Case Rep.

[18] Hurley P, Weikel C, Temeles D, Rosenberg S, Pearson R (1987). Unusual remission of Pneumocystis carinii pneumonia in a patient with the acquired immune deficiency syndrome. Am J Med.

[19] Daley CL, Iaccarino JM, Lange C (2020). Treatment of nontuberculous mycobacterial pulmonary disease: an official ATS/ERS/ESCMID/IDSA Clinical Practice Guideline. Clin Infect Dis.

[20] Kuhlman JE (1999). Imaging pulmonary disease in AIDS: state of the art. Eur Radiol.

